# Attitudes to Restrictions on the Promotion and Parental Supply of Zero Alcohol Products

**DOI:** 10.1111/dar.70123

**Published:** 2026-03-03

**Authors:** Laura Bathie, Asad Yusoff, Bella Sträuli, Michelle I. Jongenelis, Paula O'Brien, Jacquie Bowden, Aimee Brownbill, Christina Norris, Simone Pettigrew

**Affiliations:** ^1^ The George Institute for Global Health, Faculty of Medicine & Health UNSW Sydney Sydney Australia; ^2^ School of Medicine and Dentistry Griffith University Gold Coast Australia; ^3^ Melbourne Centre for Behaviour Change Melbourne School of Psychological Sciences, University of Melbourne Melbourne Australia; ^4^ Melbourne Law School University of Melbourne Melbourne Australia; ^5^ National Centre for Education and Training on Addiction, Flinders Health and Medical Research Institute Adelaide Australia; ^6^ Foundation for Alcohol Research and Education Canberra Australia

**Keywords:** advertising, children, parents, policy, zero alcohol

## Abstract

**Introduction:**

Concerns have emerged regarding the potential of zero alcohol products (ZAP) to increase youth exposure to alcohol branding and normalise consumption of alcohol‐flavoured/branded beverages. To inform future policies, this study aimed to explore attitudes to restrictions on ZAPs advertising in public spaces and parental provision of ZAPs to teenagers.

**Methods:**

The study sample comprised 3310 Australian adults who completed an online panel survey. Respondents reported level of agreement with statements about: (i) banning ZAPs advertising on public transport; (ii) banning ZAPs advertising on billboards near schools; (iii) removing ZAPs advertising from professional sport; and (iv) the acceptability of parents providing ZAPs to teenagers. Responses were analysed descriptively and via linear regression to identify demographic correlates of agreement.

**Results:**

Support for advertising restrictions was modest, with 47% of respondents agreeing with banning ZAPs advertising near schools, 33% with removing ZAPs advertising from professional sport and 31% with banning ZAPs advertising on public transport. Nearly half (45%) disagreed with parental provision of ZAPs to teenagers. Overall, older age and lower socioeconomic status were associated with small increases in support for advertising restrictions and opposition to parental supply.

**Discussion and Conclusions:**

Support for ZAPs‐related restrictions is lower than for comparable alcohol advertising policies, potentially reflecting limited awareness of ZAPs’ role in promoting alcohol brands. These findings underscore the need for public education on ZAPs‐related risks and suggest that specific messaging may be necessary to build support for regulatory interventions aimed at protecting youth.

## 1 Introduction

Zero alcohol products (ZAP) (i.e., beverages that mimic the taste and appearance of alcohol but contain no or very low alcohol content [1]), have the potential to reduce alcohol‐related harms where they are used as substitutes for alcoholic beverages [2]. However, they may also have negative consequences if they lead to ‘addition’ consumption—resulting in greater consumption of alcohol‐flavoured/branded products—either among drinkers or non‐drinkers [3]. An example is when minors who would not otherwise consume alcohol choose ZAPs. This is an issue because: (i) consumption of ZAPs could acclimatise children to the taste of alcohol; and (ii) ZAPs and their alcohol counterparts frequently share branding, resulting in the potential use of advertising and packaging of these products to promote alcohol brands to young people [4, 5].

Due to the recent popularisation of ZAPs, evidence is lacking on the nature and size of substitution and addition effects. Initial studies have yielded mixed results [3, 6, 7], suggesting the process by which these products are integrated into beverage markets may vary by cultural contexts and over time. The lack of definitive evidence has resulted in recommendations for a precautionary approach that involves ensuring ZAPs advertising complies with the same rules applied to alcohol products, especially in terms of minimising children's exposure [8]. Attitudes to such restrictive policies are unknown; this information is important because public support is a key determinant of government appetite for regulatory intervention [9].

The present study fills this evidence gap by exploring attitudes to three policies that address advertising of ZAPs in public places where children are likely to be exposed: on public transport, on billboards in close proximity to schools, and in professional sport sponsorships. The context of the study is Australia, where alcohol use is high by international standards [10], and there is current debate on the appropriateness of alcohol and ZAPs advertising in locations where children are exposed [11, 12]. Recent research shows that Australian youth automatically think of the parent company's alcohol brands when exposed to ZAPs advertising [13], reflecting concerns about the potential for ZAPs to promote alcohol brands to minors [5, 14]. ZAPs are widely advertised and appealing to young Australians [14], yet parents have been found to view them as ‘adult beverages’ that are unnecessary for youth and primarily a commercial opportunity for industry [12, 15]. Advertising placement is primarily governed by a voluntary code of conduct administered by the alcohol industry, the Alcohol Beverages Advertising Code Scheme (ABAC). ABAC specifies that alcohol and ZAPs advertising should not target children; however, it has been substantially criticised for being too lax in its requirements, determinations and enforcement actions [11, 16].

Beyond the remit of existing alcohol policy is guidance on parents' provision of ZAPs to minors, through which they may be exposed and acculturated to alcohol flavours and branding [5]. Previous Australian research indicates parental supply is a contentious issue, with a range of views held within and among different population sub‐groups [12, 14, 15]. However, these studies have been limited by small sample sizes and further research is needed to gain a broader understanding of Australians' attitudes towards parental supply of ZAPs. Advertising in public places and parental supply are likely to represent key mechanisms via which youth are exposed to ZAPs. Therefore, to obtain insights into evidence gaps relating to attitudes to restrictions on these forms of exposure and parental supply of ZAPs, the aims of the present study were to explore overall and by population sub‐groups: (i) extent of support for restrictions on ZAPs advertising in public places; and (ii) attitudes to parental supply of ZAPs to teenagers.

## 2 Methods

As part of a larger study examining responses to alcohol marketing, 3310 adult respondents (18+ years) completed an online survey during which they indicated their level of agreement with statements relating to ZAPs advertising in public places and parental supply. The advertising‐related items were ‘Advertising of zero alcohol products should not be permitted on public transport’, ‘Advertising of zero alcohol products should not be permitted on billboards outside schools’ and ‘Advertising of zero alcohol products should be removed from elite/professional sport’. To minimise negative reactance to perceived encroachment on parents' rights, the item relating to parental supply was expressed as ‘I think it's okay for parents to give zero alcohol products to their teenagers’. Responses were elicited on 5‐point agreement scales ranging from ‘Strongly disagree’ to ‘Strongly agree’. The survey was distributed via an ISO‐accredited web panel provider (Pureprofile) using age, sex and location (metropolitan/regional and state/territory jurisdiction) quotas (sample profile shown in Table 1). The study protocol was approved by a university Human Research Ethics Committee.

**TABLE 1:** Sample profile.

Demographics	Total sample *n* = 3310	
	*n*	%
Sex		
Female	1690	51
Male	1617	49
Non‐binary	3	< 1
Age, years		
18–34	952	29
35–54	1125	34
55+	1233	37
Location		
Metropolitan	2292	69
Regional/remote	1018	31
Socioeconomic position^a^		
Low (deciles 1–4)	894	27
Mid (deciles 5–8)	1308	40
High (deciles 9–10)	1108	33
Alcohol use within low‐risk guideline^b^	1307	39

^a^ Derived from postcode as per the Australian Bureau of Statistics' Socio‐Economic Index For Areas Index of Relative Disadvantage [17].

^b^ As per National Health and Medical Research Council guideline of no more than 10 standard drinks per week and no more than 4 on a single occasion [18].

Descriptive statistics (mean, standard deviation, and percentage agreement) were used to assess overall support for each statement and by sex, alcohol consumption status (within vs. exceeding the Australian drinking guideline [18], as determined by respondents' answers to quantity‐frequency measures), socioeconomic status (as assessed via postcode) and location sub‐groups (metropolitan or non‐metropolitan). A composite policy support score was subsequently calculated by averaging the mean score of each policy support question, overall and by participant characteristics. Following this, a linear regression analysis was conducted to explore associations between participant characteristics and greater policy support. The dependent variables included in this analysis were: age, gender, socioeconomic status, location and adherence to the Australian drinking guidelines.

## 3 Results

Average scores were around 3 on the 5‐point agreement scales, indicating neutral levels of support overall and for most sub‐groups (Table 2). Active support (selecting ‘Agree’ or ‘Strongly agree’) was highest for banning ZAPs advertising on billboards located close to schools (47%) and lower for removing ZAPs from elite/professional sport (33%) and banning ZAPs advertising on public transport (31%). Almost half (45%) of respondents did not agree with parents providing ZAPs to their teenagers. Active disagreement (selecting ‘Disagree’ or ‘Strongly disagree’) was notably higher among those drinking within the alcohol guideline, females, older respondents, non‐metropolitan residents and those residing in low socioeconomic areas. Table S1 provides detailed results for agree, disagree and neutral responses for each item.

**TABLE 2:** Descriptive results.

Policies	Advertising of zero alcohol products should not be permitted on public transport		Advertising of zero alcohol products should not be permitted on billboards outside schools		Advertising of zero alcohol products should be removed from elite/professional sport		It is not okay for parents to give zero alcohol products to their teenagers^c^		Composite mean score
	M (SD)	% agree^b^	M (SD)	% agree^b^	M (SD)	% agree^b^	M (SD)	% agree^b^	
Total sample (*n* = 3310)	2.97 (1.13)	31	3.33 (1.18)	47	3.02 (1.15)	33	3.36 (1.12)	45	3.17
Alcohol consumption within guideline^a^ (*n* = 1307)	2.95 (1.12)	29	3.39 (1.20)	49	3.00 (1.15)	32	3.55 (1.03)	52	3.22
Alcohol consumption exceeding guideline^a^ (*n* = 2003)	2.98 (1.14)	32	3.29 (1.16)	45	3.04 (1.16)	34	3.23 (1.15)	40	3.14
Females (*n* = 1690)	2.93 (1.13)	29	3.32 (1.19)	46	3.02 (1.17)	33	3.43 (1.11)	48	3.18
Males (*n* = 1617)	3.01 (1.13)	33	3.34 (1.16)	47	3.02 (1.14)	33	3.28 (1.11)	42	3.16
18–34 years (*n* = 952)	2.89 (1.16)	31	3.21 (1.19)	45	2.93 (1.17)	33	3.03 (1.15)	35	3.02
35–54 years (*n* = 1125)	3.05 (1.16)	34	3.33 (1.19)	46	3.10 (1.16)	36	3.40 (1.14)	46	3.22
55+ years (*n* = 1233)	2.96 (1.08)	28	3.42 (1.15)	49	3.03 (1.13)	31	3.57 (1.00)	52	3.25
Metropolitan (*n* = 2292)	2.99 (1.14)	32	3.32 (1.17)	47	3.03 (1.15)	34	3.33 (1.13)	44	3.17
Non‐metropolitan (*n* = 1018)	2.92 (1.13)	28	3.36 (1.19)	47	3.00 (1.15)	31	3.41 (1.08)	48	3.17
Low socioeconomic position^d^ (*n* = 894)	2.99 (1.15)	31	3.41 (1.19)	50	3.06 (1.17)	34	3.43 (1.09)	48	3.22
Mid socioeconomic position^d^ (*n* = 1308)	2.98 (1.12)	30	3.31 (1.17)	46	3.01 (1.15)	32	3.40 (1.11)	46	3.12
High socioeconomic position^d^ (*n* = 1108)	2.95 (1.14)	32	3.29 (1.17)	45	3.01 (1.15)	34	3.25 (1.13)	40	3.13

^a^ As per National Health and Medical Research Council guideline of no more than 10 standard drinks per week and no more than 4 on a single occasion [18].

^b^ Proportion selecting ‘Agree’ (4) or ‘Strongly agree’ (5) on a 5‐point agreement scale.

^c^ Original item (‘It's not okay for parents to give zero alcohol products to their teenagers) reverse coded: ‘Disagree’ (4), ‘Strongly disagree’ (5).

^d^ Derived from postcode as per the Australian Bureau of Statistics' Socio‐Economic Index For Areas Index of Relative Disadvantage [17].

The regression results are displayed in Table 3. Age (positive) and socioeconomic status (negative) had small but significant associations with the composite score, on which higher values reflected greater support for ZAPs advertising restrictions and stronger disagreement with parental provision of ZAPs.

**TABLE 3:** Multiple linear regression results for composite agreement score (*n* = 3307).

	Standardised coefficient (SE)	95% confidence interval	*p*
Female	0.01 (0.02)	[−0.02–0.05]	0.408
Age (continuous)	0.09 (0.02)	[0.05–0.13]	0.000**
Socioeconomic status^a^ (ordinal)			
Mid	−0.03 (0.02)	[−0.05–0.03]	0.554
High	−0.06 (0.02)	[−0.11–0.02]	0.006*
Metropolitan location	0.03 (0.02)	[−0.01–0.07]	0.104
Exceeds alcohol consumption guideline^b^	−0.03 (0.02)	[−0.07–0.00]	0.065

^a^ Derived from postcode as per the Australian Bureau of Statistics' Socio‐Economic Index For Areas Index of Relative Disadvantage [17].

^b^ As per National Health and Medical Research Council guideline of no more than 10 standard drinks per week and no more than 4 on a single occasion [18].

* *p* < 0.05.

** *p* < 0.001.

## 4 Discussion

The identified levels of support for restrictions on ZAPs advertising were modest and lower than those found in prior research relating to alcohol advertising [19]. For example, 54% of previously sampled Australian adults agreed that alcohol advertising should not be permitted on government‐owned infrastructure, including public transport (compared to 31% for ZAPs in this study), and 50% agreed that alcohol advertising should be removed from professional sport (compared to 33%) [19].

Almost half the present sample (45%) disagreed with parents providing ZAPs to their teenagers. This outcome reflects prior Australian studies indicating considerable variation in both parents' and the general public's perceptions of the appropriateness of parental supply of ZAPs [12, 14, 15]. Overall, these results may suggest greater tolerance for advertising and provision of ZAPs compared to alcohol products, reflecting inadequate community understanding about the potential use of ZAPs as a form of alibi marketing to promote alcohol brands, including to children [4].

The regression results showed that older age and lower socioeconomic status were associated with a small but statistically significant increase in the composite score, indicating greater support for advertising restrictions and parental non‐supply. Sex, location, and alcohol consumption category differences were not significant in the model. The age outcome is consistent with previous research examining Australians' support for more stringent alcohol policy [19], while the socioeconomic outcome is in the opposite direction [19]. The latter outcome may be related to consumption of ZAPs being more common among higher income consumers [20], resulting in greater acceptance of the promotion and provision of these products.

The results of this study highlight the importance of educating the public about the potential harms associated with ZAPs to increase support for recommended harm‐reduction strategies. In particular, there appears to be a need for greater societal understanding of the risks associated with increasing youth exposure to alcohol branding and alcohol‐flavoured products that have the potential to further habituate minors into an alcohol culture [5]. The World Health Organization has emphasised the importance of informing communities about these risks, noting that community support is a critical part of the advocacy process that encourages governments to implement appropriate strategies to protect young people [8].

A primary limitation of this study was the focus on a small number of actions designed to reduce any harms associated with ZAPs. Further research is needed to explore attitudes to a broader range of recommended policies (e.g., restricting promotion via other media channels such as social, broadcast and print media, banning any ZAPs advertising that associates the products with the parent alcohol brand and establishing rules for locations where ZAPs can be sold [8]). Future research should also assess interactions between ZAP exposure, ZAP use, and alcohol use among young people to inform public education activities. Second, support for policies restricting parental supply of ZAPs was not directly measured due to the lack of current laws prohibiting parental supply of alcohol and the sensitivity of this issue. Instead, general attitude to parental supply of ZAPs was explored to provide relevant insights. Third, a sample skew towards higher socioeconomic status individuals constrains the generalisability of the results. Finally, this research was confined to Australia, and similar work should be conducted in other countries to inform the development of education programs and regulatory frameworks that limit young people's exposure to alcohol brand advertising.

## Author Contributions

Each author certifies that their contribution to this work meets the standards of the International Committee of Medical Journal Editors.

## Funding

This study was funded by the National Health and Medical Research Council (APP2021186). S.P. is funded by NHMRC Investigator Grant (#2034602).

## Conflicts of Interest

The authors declare no conflicts of interest.

## Supporting information


**Data S1:** dar70123‐sup‐0001‐Supinfo.docx.

## Data Availability

The data that support the findings of this study are available from the corresponding author upon reasonable request.
