# Supplementary material for: Attitudes to Restrictions on the Promotion and Parental Supply of Zero Alcohol Products
Source: Drug Alcohol Rev. 2026 Mar 3;45(3):e70123. doi: 10.1111/dar.70123 (PMC12956468; doi:10.1111/dar.70123)
Supplement: Supplementary file 1 — Data S1: dar70123‐sup‐0001‐Supinfo.docx. [file DAR-45-0-s001.docx]

**Supporting Information**

*Table S1. Responses to ZAPs restrictions and parental supply, by demographics*

| Policies | Advertising of zero alcohol products should not be permitted on public transport | | | Advertising of zero alcohol products should not be permitted on billboards outside schools | | | Advertising of zero alcohol products should be removed from elite/professional sport | | | It is not okay for parents to give zero alcohol products to their teenagers# | | |
| --- | --- | --- | --- | --- | --- | --- | --- | --- | --- | --- | --- | --- |
|  | %  Agree | % Neutral | % Disagree | %  Agree | % Neutral | % Disagree | %  Agree | % Neutral | % Disagree | %  Agree | % Neutral | % Disagree |
| Total sample (n = 3310) | 31 | 34 | 35 | 47 | 28 | 25 | 33 | 34 | 33 | 45 | 31 | 24 |
| Alcohol consumption within guideline^ (n = 1307) | 29 | 35 | 36 | 49 | 26 | 25 | 32 | 33 | 35 | 52 | 30 | 17 |
| Alcohol consumption exceeding guideline^ (n = 2003) | 32 | 33 | 35 | 45 | 30 | 25 | 34 | 34 | 32 | 40 | 32 | 28 |
| Females (n = 1690) | 29 | 34 | 37 | 46 | 28 | 26 | 33 | 33 | 34 | 48 | 30 | 22 |
| Males (n = 1617) | 33 | 34 | 34 | 47 | 29 | 24 | 33 | 34 | 32 | 42 | 33 | 26 |
| 18-34 years (n = 952) | 31 | 30 | 39 | 45 | 26 | 29 | 33 | 31 | 37 | 35 | 29 | 36 |
| 35-54 years (n = 1125) | 34 | 34 | 33 | 46 | 30 | 24 | 36 | 33 | 31 | 46 | 31 | 23 |
| 55+ years (n = 1233) | 28 | 38 | 34 | 49 | 29 | 22 | 31 | 36 | 33 | 52 | 34 | 15 |
| Metropolitan (n = 2292) | 32 | 33 | 35 | 47 | 29 | 25 | 34 | 33 | 33 | 44 | 32 | 24 |
| Non-metropolitan (n = 1018) | 28 | 36 | 36 | 47 | 28 | 25 | 31 | 34 | 34 | 48 | 31 | 22 |
| Low socioeconomic position~ (n = 894) | 31 | 35 | 34 | 50 | 27 | 23 | 34 | 35 | 31 | 48 | 30 | 22 |
| Mid socioeconomic position~  (n = 1308) | 30 | 36 | 34 | 46 | 30 | 25 | 32 | 34 | 34 | 46 | 32 | 21 |
| High socioeconomic position~ (n = 1108) | 32 | 31 | 37 | 45 | 28 | 27 | 34 | 32 | 34 | 40 | 31 | 27 |

Note: ‘Agree’ = proportion selecting ‘Agree’ (4) or ‘Strongly agree’ (5); ‘Neutral’ = proportion selecting ‘Neither agree nor disagree’ (3); ‘Disagree’ = proportion selecting ‘Disagree’ (2) or ‘Strongly disagree’ (1) on a 5-point agreement scale.

^ As per National Health and Medical Research Council guideline of no more than 10 standard drinks per week and no more than 4 on a single occasion [1].

# Original item (‘It’s not okay for parents to give zero alcohol products to their teenagers) reverse coded: ‘Disagree’ (4), ‘Strongly disagree’ (5).

~ Derived from postcode as per the Australian Bureau of Statistics’ Socio-Economic Index For Areas Index of Relative Disadvantage [2].

**References**

1. National Health and Medical Research Council. Australian Guidelines to Reduce Health Risks from Drinking Alcohol. Canberra; 2020.
2. Australian Bureau of Statistics. Socio-economic indexes for areas (SEIFA) (2023). Available at: https://www.abs.gov.au/statistics/people/people-and-communities/socio-economic-indexes-areas-seifa-australia/latest-release (accessed August 2025).
